# The influence of protocol design on the identification of ventilatory thresholds and the attainment of peak physiological responses during synchronous arm crank ergometry in able-bodied participants

**DOI:** 10.1007/s00421-019-04211-9

**Published:** 2019-08-21

**Authors:** Ingrid Kouwijzer, Mitch Valize, Linda J. M. Valent, Paul Grandjean Perrenod Comtesse, Lucas H. V. van der Woude, Sonja de Groot

**Affiliations:** 1 Research and Development, Heliomare Rehabilitation Center, Wijk aan Zee, The Netherlands; 2 Center for Human Movement Sciences, University of Groningen, University Medical Center Groningen, Groningen, The Netherlands; 3 Amsterdam Rehabilitation Research Center | Reade, Amsterdam, The Netherlands; 4 Faculty of Health Medicine and Life Sciences, Maastricht University, Maastricht, The Netherlands; 5 Orbis Sport Sports Medical Center, Sittard-Geleen, The Netherlands; 6 Adelante Rehabilitation Center, Hoensbroek, The Netherlands; 7 Center for Rehabilitation, University of Groningen, University Medical Center Groningen, Groningen, The Netherlands

**Keywords:** Protocol type, Graded exercise test, Physical capacity, Ventilatory thresholds, Training

## Abstract

**Purpose:**

To examine the effects of stage duration on power output (PO), oxygen uptake (*V*O_2_), and heart rate (HR) at peak level and ventilatory thresholds during synchronous arm crank ergometry.

**Methods:**

Nineteen healthy participants completed a ramp, 1-min stepwise, and 3-min stepwise graded arm crank exercise test. PO, *V*O_2,_ and HR at the first and second ventilatory threshold (VT1, VT2) and peak level were compared among the protocols: a repeated measures analysis of variance was performed to test for systematic differences, while intraclass correlation coefficients (ICC) and Bland–Altman plots were calculated to determine relative and absolute agreement.

**Results:**

Systematic differences among the protocols were found for PO at VT1, VT2, and peak level. At peak level, PO differed significantly among all protocols (ramp: 115 ± 37 W; 1-min stepwise: 108 ± 34 W; 3-min stepwise: 94 ± 31 W, *p* ≤ 0.01). No systematic differences for HR or *V*O_2_ were found among the protocols. VT1 and VT2 were identified at 52% and 74% of *V*O_2_peak, respectively. The relative agreement among protocols varied (ICC 0.02–0.97), while absolute agreement was low with small-to-large systematic error and large random error.

**Conclusions:**

PO at VTs and peak level was significantly higher in short-stage protocols compared with the 3-min stepwise protocol, whereas HR and *V*O_2_ showed no differences. Therefore, training zones based on PO determined in short-stage protocols might give an overestimation. Moreover, due to large random error in HR at VTs between the protocols, it is recommended that different protocols should not be used interchangeably within individuals.

## Introduction

Handcycling is a rapidly growing sport for people with disabilities worldwide, especially in persons with spinal cord injury (SCI), muscular disease, or leg amputation (Abel et al. 2010). People have turned to handcycling as a means to improve their physical capacity during or after rehabilitation (Hoekstra et al. 2017; Valent et al. 2009). To promote handcycling as an exercise mode in The Netherlands, an annual handcycle event called the HandbikeBattle is organised in Austria since 2013 (De Groot et al. 2014).

To become physically fit or to prepare optimally for such an event, a valid and reliable individualised training scheme is necessary. The intensity of the aerobic endurance training in this training scheme is based on the cardiorespiratory fitness of the individual, measured during a graded exercise test (GXT). Results of the GXT are used to develop individualised schemes based on percentages of peak power output (POpeak) or peak heart rate (HRpeak), or based on training zones delineated by power output (PO) or heart rate (HR) at ventilatory thresholds (VTs) (Lucia et al. 1999; Meyer et al. 2005; Seiler and Kjerland 2006; Wolpern et al. 2015). These VTs provide boundaries to set individualized training zones: zone 1 at low intensity [below the first ventilatory threshold (VT1)], zone 2 at moderate intensity [between VT1 and the second ventilatory threshold (VT2)], and zone 3 at high intensity (above VT2) (Meyer et al. 2005; Seiler and Kjerland 2006). Over the years, several GXT protocol designs with varying stage durations have been employed. For example, workload increases at set intensities following a defined interval of time as a series of “steps” in a stepwise protocol, or workload increases in a smooth linear way in a ramp protocol (Bentley and McNaughton 2003; Bishop et al. 1998; Gullestad et al. 1997; Larson et al. 2015; Maher and Cowan 2016; Roffey et al. 2007; Smith et al. 2004, 2006; Zuniga et al. 2012). It is, however, not entirely known what the effects are of these different types of protocols and stage durations on outcome measures such as oxygen uptake (*V*O_2_), PO and HR, at both peak exercise and VTs during synchronous arm exercise.

In asynchronous arm cranking, two studies investigated effects of stage duration on peak physiological responses. Smith et al. (2004) compared a 2-min stepwise protocol with a ramp protocol in able-bodied participants (*N* = 14), whereas Maher and Cowan (2016) compared a 1-min with a 3-min stepwise protocol in individuals with SCI (*N* = 38). The protocols were designed in such a way that patterns of work rate increase, external work, and test duration was comparable between protocols. Both studies found no significant differences in *V*O_2_peak, HRpeak, and POpeak between the different protocols. In addition, Smith et al. (2006) compared two ramp protocols with different ramp slopes. While *V*O_2_peak and HRpeak were unaffected, they found a significantly higher POpeak and shorter test duration in the protocol with a steeper ramp slope.

In able-bodied cycling, effects from protocols with different stage durations are studied widely. In general, POpeak was higher in ramp protocols or protocols with short-stage duration, compared with protocols with longer stage duration (Amann et al. 2004; Bentley and McNaughton 2003; Bishop et al. 1998; Gullestad et al. 1997; Larson et al. 2015; Roffey et al. 2007; Zuniga et al. 2012). In the tests with longer stage duration, total test duration was also longer. Peak oxygen uptake (*V*O_2_peak) was not significantly different between protocols (Bentley and McNaughton 2003; Bishop et al. 1998; Larson et al. 2015; Roffey et al. 2007; Zuniga et al. 2012), or was higher in protocols with longer stage duration (Gullestad et al. 1997). HRpeak was not significantly different between protocols (Bentley and McNaughton 2003; Gullestad et al. 1997; Zuniga et al. 2012), or was higher in protocols with longer stage duration (Bishop et al. 1998; Larson et al. 2015; Roffey et al. 2007). Bentley and McNaughton (2003) found that *V*O_2_ and HR at VT1 were not significantly different between a 1-min and a 3-min stepwise protocol, whereas PO at VT1 was significantly higher in the 1-min stepwise protocol.

The effects of these different types of protocols and stage durations on *V*O_2_, PO, and HR, at both peak level and VTs, have previously not been studied with synchronous arm exercise. Traditionally, protocols with longer stage duration are executed to determine submaximal responses and the position of thresholds. However, in the last years, supported by technological innovations, ramp protocols became popular, also to detect VTs (Mezzani 2017). The advantage of ramp protocols is that the work changes over time are not affected by protocol steps, leading to linear physiological responses (Boone and Bourgois 2012; Mezzani 2017; Myers and Bellin 2000). The consequence is, however, that the *V*O_2_ response is specific to the non-steady-state character of the protocol. Typically, the *V*O_2_ response shows a lag to the metabolic demand (i.e., mean response time) (Boone and Bourgois 2012). The measured *V*O_2_ at any work rate will underestimate the steady-state *V*O_2_ at that work rate (Davis et al. 1982; Smith et al. 2006). Since VTs are often used to set up training schemes (Meyer et al. 2005), it is of importance to know whether the position of VTs is affected by the used test protocol with corresponding test duration and step size. Therefore, the aim of this study was to examine the effects of stage duration with a ramp protocol, 1-min stepwise protocol, and 3-min stepwise protocol on PO, *V*O_2,_ and HR at both peak level and at VT1 and VT2 during synchronous arm crank ergometry. We hypothesized that *V*O_2_ and HR at VTs and peak level will not be affected by stage duration, whereas PO at VTs and peak level will be higher within short-stage protocols, compared with the 3-min stepwise protocol.

## Materials and methods

### Participants

Nineteen able-bodied individuals were recruited to participate in the study: nine men/ten women, age (mean ± standard deviation) 30 ± 10 years with range: 21–58 years, body mass: 71.6 ± 9.9 kg, height: 1.78 ± 0.07 m. All participants were healthy and physically active. They participated recreationally in sports such as fitness, soccer, and running with an average of four hours a week. They were non-specifically arm trained and had no experience with GXT on an arm crank ergometer. They had no restrictions or injuries of the upper extremities, and did not suffer from chronic diseases, such as heart or lung disease, diabetes, or obesity. Before the start of the test, participants were medically screened using the Physical Activity Readiness Questionnaire (PAR-Q) (Chisholm et al. 1975). Informed consent was obtained from all individual participants included in the study. The study was approved by the Ethics Committee of the Center for Human Movement Sciences, University Medical Center Groningen, University of Groningen, The Netherlands.

### Test procedure

All participants performed three GXTs in synchronous cranking mode: one with a ramp protocol, one with a 1-min stepwise protocol, and one with a 3-min stepwise protocol. The order of the tests was counter-balanced and each test was separated by at least 3 days with a maximum of 7 days. The three tests were executed at the same time of the day within participants, but varied between participants. Participants were required to abstain from alcohol, caffeine, and smoking 12 h before testing and from strenuous physical activity for 24 h.

All tests were conducted on an electrically braked arm crank ergometer (Lode Angio, Groningen, The Netherlands). The ergometer was wall-mounted using a height-adjustable bracket. Participants were seated, so that the axis of rotation of the arm crank was at the same height as the axis of rotation of the shoulder joint, and positioned at a comfortable distance from the ergometer allowing for a slight bend (15°–20°) of the participant’s elbow at the furthest point of the range of movement (Mossberg et al. 1999; Smith et al. 2004). Participants were required to sit back firmly in the chair to maintain a standardized position, and were instructed to keep their feet in front of them at shoulder width and flat on the floor throughout each test.

For the ramp protocol and the 1-min stepwise protocol, the aim was to develop a protocol with a test duration between 8 and 12 min (Buchfuhrer et al. 1983), and a longer test duration with at least six steps (18 min) for the 3-min stepwise protocol (Amann et al. 2004; Bishop et al. 1998). The starting workload and step size or ramp slope were based on pilot experiments and previous literature (Hopman et al. 1995; Smith et al. 2004; Widman et al. 2007). The pilot experiments were conducted in individuals comparable to the studied population and pilot results were not included in the present study. All tests started with a resting period of 2 min, followed by a warm-up of 5 min on 20 W. For male participants, the test protocols were as follows: ramp: start at 0 W with increments 1 W/5 s (i.e., equivalent to 12 W/min); 1-min stepwise: start at 10 W with increments 12 W/min; and 3-min stepwise: start at 10 W with increments 20 W/3 min. For female participants, the test protocols were as follows: ramp: start at 0 W with increments 1 W/7.5 s (i.e., equivalent to 8 W/min); 1-min stepwise: start at 10 W with increments 8 W/min; and 3-min stepwise: start at 10 W with increments 14 W/3 min. After all tests, a cool-down of 5 min was performed on 20 W. During the test, participants were instructed to maintain a crank rate of 60–80 revolutions per minute (RPM). Criteria to stop the test were volitional exhaustion or failure in keeping a constant cadence above 60 RPM. Verbal encouragements were given towards the end of the test. At the end of the test, ratings of perceived exertion (RPE) were recorded using the 10-point Borg scale (Borg 1982).

### Determination of peak physiological responses

Respiratory data, including *V*O_2_, carbon dioxide production (*V*CO_2_), minute ventilation (VE), and respiratory exchange ratio (RER), were collected and analysed per 10 s by mixing-chamber technique using the Cortex (Cortex, CORTEX Biophysik GmbH, Germany). The equipment was calibrated before each test. Criteria for a peak test were: RPE ≥ 8 at the end of the test and RERpeak ≥ 1.10 (Mezzani 2017). For all three protocols, *V*O_2_peak, *V*CO_2_peak, VE, and RERpeak were defined as the highest 30-s average value of *V*O_2,_*V*CO_2,_ VE, and RER, respectively. HR was recorded continuously from rest through recovery using a wireless chest strap monitor (Polar T31, Finland). HRpeak was defined as the highest 10-s average value achieved. In the 1-min stepwise protocol, POpeak was defined as the last completed PO step, plus ½ times the PO increment for each 30-s block in the non-completed PO step. In the 3-min stepwise protocol, POpeak was defined as the last completed PO step, plus 1/6 times the PO increment for each 30-s block in the non-completed PO step (Kuipers et al. 1985). In the ramp protocol, POpeak was defined as the highest 10-s PO achieved at the end of the test. In addition, total accumulated work done (TWD in kJ) was calculated, as described in the previous literature (Smith et al. 2004; Zuniga et al. 2012).

### Determination of ventilatory thresholds

For the three protocols, all data were represented in plots as described by Wasserman et al. (2012). A combination of two plots was examined to determine VT1: (1) *V*CO_2_ vs *V*O_2_ and (2) the ventilatory equivalents of oxygen (VE/*V*O_2_) and carbon dioxide (VE/*V*CO_2_) vs time. VT1 was defined as an increase in slope of more than 1 in the first plot (V-slope method) (Beaver et al. 1986; Gaskill et al. 2001; Leicht et al. 2014; Meyer et al. 2005), and/or as the first sustained rise in VE/*V*O_2_ without a concomitant increase in VE/*V*CO_2_ in the second plot (Ventilatory Equivalents method) (Beaver et al. 1986; Binder et al. 2008; Caiozzo et al. 1982; Gaskill et al. 2001; Leicht et al. 2014).

A combination of three plots was examined to determine VT2: (1) VE vs *V*CO_2_; (2) VE/*V*O_2_ and VE/*V*CO_2_ vs time; and (3) *V*CO_2_ vs *V*O_2_. VT2 was defined as the inflection in the VE vs *V*CO_2_ slope in the first plot (Binder et al. 2008; Meyer et al. 2005; Mezzani et al. 2012), and/or the first systematic increase in VE/*V*CO_2_ (Ventilatory Equivalents method) in the second plot (Binder et al. 2008; Meyer et al. 2005; Mezzani et al. 2012), and/or as a second increase in slope in the third plot with *V*CO_2_ vs *V*O_2_ (Aunola and Rusko 1984; Meyer et al. 2005).

Two trained researchers independently examined the plots visually to determine both VTs. Thereafter, results were compared. The interrater reliability (intraclass correlation coefficient (ICC)) of the determined VTs was 0.93 (95% CI 0.84–0.97) for VT1 (based on *N* = 48) and 0.94 (95% CI 0.90–0.97) for VT2 (based on *N* = 48). On average, there was a 4.1% and 0.6% difference between raters for VT1 and VT2, respectively. When the raters did not have the exact same point in time for a VT (*N* = 65), they examined the plots together to come to a mutually agreed VT. This value was then used for further analysis.

Thereafter, *V*O_2_ and HR at the VTs were determined as the 10-s value on that point in time. PO at the VTs was determined as follows: the last completed PO step, plus ½ times the PO increment for each 30-s block in the non-completed PO step for the 1-min stepwise protocol; the last completed PO step, plus 1/6 times the PO increment for each 30-s block in the non-completed PO step for the 3-min stepwise protocol; and the PO achieved at that specific point in time for the ramp protocol. TWD at the VTs was calculated as the accumulated work [PO (W) × time (s)] at that point in time. The relative values of *V*O_2_, HR, PO, and TWD at both VTs were calculated as the absolute value at that VT divided by the peak value of that respective outcome measure (i.e., *V*O_2_peak, HRpeak, POpeak, and TWD at peak).

### Statistical analyses

Data were analysed using IBM SPSS Statistics 24 (IBM SPSS Statistics 24, SPSS, Inc, Chicago, IL, USA). The data were tested for normality using the Kolmogorov–Smirnov test with Lilliefors Significance Correction, the Shapiro–Wilk test, and *z* scores for skewness and kurtosis. The peak physiological responses and test duration, and the *V*O_2_, HR, PO and TWD at VT1 and VT2, were compared among protocols. To test for systematic differences, a repeated measures analysis of variance (ANOVA) was performed. Mauchly’s test was used to test the assumption of sphericity. A Bonferroni post-hoc test for multiple comparisons was used for pairwise comparisons. Due to the potential risk of bias, imputation of data was not considered. Cohen’s *d* effect sizes were calculated and were evaluated according to Hopkins (2002) as trivial (0–0.19), small (0.20–0.59), moderate (0.60–1.19), large (1.20–1.99), or very large (≥ 2.00). The ICC was used to measure relative agreement (2.1: two-way random, absolute agreement, and single measures), and Bland–Altman plots with 95% limits of agreement (LoA) were used to measure absolute agreement (systematic error and random error) (Bland and Altman 1986). The following interpretation was used for the ICC 0.00–0.25, little to no correlation; 0.26–0.49, low correlation; 0.50–0.69, moderate correlation; 0.70–0.89, high correlation; and 0.90–1.00, very high correlation (Munro 2004). Values were considered significant at *p* < 0.05, and data were reported as mean (± SD) unless otherwise stated.

## Results

All participants completed all tests successfully resulting in a total of 57 tests. In total, 101 out of 114 (89%) VTs could be determined. Of the 13 undetermined VTs, 2 were related to the ramp protocol, 5 to the 1-min stepwise protocol, and 6 to the 3-min stepwise protocol. Eight were VT1 and 5 were VT2 of these 13 undetermined VTs. Outcomes were normally distributed. Peak values and threshold characteristics are shown in Table 1. RPE at peak was on average 10 ± 0 for the ramp and 3-min stepwise protocol, and 10 ± 1 for the 1-min stepwise protocol.

**Table 1:** Descriptives and results of the repeated measures ANOVA for the ramp, 1-min and 3-min protocol

	Ramp		1-min		3-min		ANOVA			Ramp vs 1 min		Ramp vs 3 min		1 min vs 3 min	
	*N*	*M* ± SD	*N*	*M* ± SD	*N*	*M* ± SD	*N*	*F*	*p* value	*p* value	Cohen’s *d*	*p* value	Cohen’s *d*	*p* value	Cohen’s *d*
Peak values															
POpeak (W)	19	115 ± 37	19	108 ± 35	19	94 ± 31	19	71.68	**< 0.01**	**< 0.01**	0.19	**< 0.01**	0.62	**< 0.01**	0.42
*V*O_2_peak (L/min)	19	1.95 ± 0.56	19	1.89 ± 0.56	19	1.99 ± 0.58	19	1.38	0.26	1.00	0.09	1.00	0.07	0.32	0.17
*V*CO_2_peak (L/min)	19	2.67 ± 0.79	19	2.64 ± 0.81	19	2.59 ± 0.72	19	0.56	0.58	1.00	0.04	0.65	0.11	1.00	0.07
VE (L/min)	19	100 ± 33	19	101 ± 34	19	102 ± 32	19	0.17	0.84	1.00	0.03	1.00	0.06	1.00	0.03
HRpeak (bpm)	19	168 ± 17	19	170 ± 16	19	171 ± 15	19	1.06	0.36	0.88	0.14	0.51	0.16	1.00	0.02
RERpeak	19	1.42 ± 0.18	19	1.43 ± 0.19	19	1.36 ± 0.19	19	2.76	0.08	1.00	0.09	0.44	0.31	0.10	0.39
Test duration (min)	19	11.5 ± 2.1	19	10.8 ± 2.4	19	17.8 ± 3.8	19	260.48	**< 0.01**	**< 0.01**	0.31	**< 0.01**	2.06	**< 0.01**	2.21
Total work done (kJ)	19	41 ± 20	19	40 ± 19	19	59 ± 29	19	48.9	**< 0.01**	1.00	0.05	**< 0.01**	0.72	**< 0.01**	0.78
Ventilatory thresholds															
PO at VT1 (W)	18	51 ± 22	16	44 ± 20	15	36 ± 14	13	4.20	**0.03**	1.00	0.33	**0.047**	0.81	0.19	0.46
% of POpeak	18	43 ± 8	16	40 ± 10	15	39 ± 8	13	0.46	0.63	1.00	0.33	1.00	0.50	1.00	0.11
PO at VT2 (W)	18	80 ± 22	17	75 ± 23	17	61 ± 18	15	15.13	**< 0.01**	0.19	0.22	**< 0.01**	0.95	**0.01**	0.68
% of POpeak	18	70 ± 10	17	68 ± 9	17	63 ± 12	15	2.38	0.14	1.00	0.21	0.36	0.63	0.41	0.57
*V*O_2_ at VT1 (L/min)	18	0.98 ± 0.36	16	1.00 ± 0.33	15	1.01 ± 0.25	13	0.48	0.63	1.00	0.08	0.74	0.26	1.00	0.14
% of VO_2_peak	18	51 ± 13	16	52 ± 8	15	52 ± 9	13	0.39	0.68	1.00	0.20	1.00	0.27	1.00	0.07
*V*O_2_ at VT2 (L/min)	18	1.45 ± 0.36	17	1.51 ± 0.47	17	1.38 ± 0.31	15	2.14	0.16	0.69	0.18	0.65	0.22	0.40	0.37
% of VO_2_peak	18	75 ± 10	17	78 ± 11	17	70 ± 14	15	6.26	**0.01**	0.20	0.39	0.14	0.49	**0.04**	0.85
HR at VT1 (bpm)	18	110 ± 21	16	114 ± 22	15	115 ± 23	13	0.12	0.89	1.00	0.07	1.00	0.11	1.00	0.03
% of HRpeak	18	65 ± 8	16	67 ± 9	15	66 ± 9	13	0.04	0.97	1.00	0.07	1.00	0.07	1.00	0.00
HR at VT2 (bpm)	18	140 ± 18	17	143 ± 17	17	136 ± 24	15	1.73	0.20	0.45	0.34	1.00	0.11	0.43	0.40
% of HRpeak	18	82 ± 8	17	84 ± 6	17	79 ± 10	15	2.57	0.10	0.57	0.36	0.71	0.30	0.25	0.64
TWD at VT1 (kJ)	18	8 *±* 6	16	8 ± 6	15	12 *±* 8	13	5.35	**0.01**	1.00	0.00	0.06	0.56	0.05	0.56
% of TWD at peak	18	19 ± 6	16	20 ± 9	15	21 ± 6	13	0.75	0.48	1.00	0.13	0.62	0.33	1.00	0.13
TWD at VT2 (kJ)	18	20 *±* 9	17	20 *±* 10	17	27 *±* 12	15	4.74	**0.04**	1.00	0.00	0.09	0.66	0.18	0.63
% of TWD at peak	18	50 ± 13	17	50 ± 12	17	46 ± 15	15	1.34	0.27	0.43	0.00	1.00	0.28	0.57	0.29

*POpeak* peak power output, *VO*_*2*_*peak* peak oxygen uptake, *VCO*_*2*_*peak* peak carbon dioxide production, *VE* minute ventilation, *HRpeak* peak heart rate, *RERpeak* peak respiratory exchange ratio, *VT1* first ventilatory threshold, *VT2* second ventilatory threshold, *TWD* total work done, *FF* (Fischer)-statistic of within-subject effects

### Systematic differences among test protocols

Results of the repeated measures ANOVA are shown in Table 1. At peak level, *V*O_2_, RER, and HR were not significantly different among protocols. POpeak differed significantly among all three protocols, with the highest value for the ramp protocol (115 ± 37 W), followed by the 1-min stepwise (108 ± 35 W) and 3-min stepwise protocol (94 ± 31 W). Test duration differed significantly among all three protocols, with the shortest test duration for the 1-min stepwise protocol (10.8 ± 2.4 min), followed by the ramp protocol (11.5 ± 2.1 min) and 3-min stepwise protocol (17.8 ± 3.8 min). TWD was significantly lower for the ramp (41 ± 20 kJ) compared with the 3-min stepwise protocol (59 ± 29 kJ) and for the 1-min stepwise (40 ± 19 kJ) compared with the 3-min stepwise protocol.

At both VTs, absolute values of *V*O_2_ were not significantly different among protocols. The relative *V*O_2_ as a percentage of *V*O_2_peak at VT2 was significantly higher for the 1-min stepwise protocol compared with the 3-min stepwise protocol. Absolute and relative values of HR at VT1 and VT2 were not significantly different among protocols. At VT1, PO was significantly higher for the ramp protocol (51 ± 22 W) compared with the 3-min stepwise protocol (36 ± 14 W). At VT2, PO was significantly higher for the ramp (80 ± 22 W) compared with the 3-min stepwise protocol (61 ± 18 W) and for the 1-min stepwise (75 ± 23 W) compared with the 3-min stepwise protocol. The relative PO as a percentage of POpeak, at both VT1 and VT2, was not significantly different among protocols. Absolute and relative values of TWD at VT1 and VT2 were not significantly different among protocols.

### Agreement among test protocols

The relative agreement varied (Table 2). Twelve percent of correlations was very high (ICC ≥ 0.90), 24% of correlations was high (ICC ≥ 0.70), whereas 64% was moderate or less (ICC ≤ 0.69).

**Table 2:** Relative agreement at peak level and thresholds during arm crank testing for the ramp, 1-min and 3-min protocol

	ICC (95% CI)		ICC (95% CI)		ICC (95% CI)	
	*N*	Ramp vs 1 min	*N*	Ramp vs 3- min	*N*	1 min vs 3 min
Peak values						
POpeak (W)	19	0.97 (0.77–0.99)*	19	0.82 (− 0.05 to 0.96)*	19	0.90 (− 0.01 to 0.98)*
*V*O_2_peak (L/min)	19	0.88 (0.71–0.95)*	19	0.93 (0.83–0.97)*	19	0.90 (0.76–0.96)*
*V*CO_2_peak (L/min)	19	0.91 (0.79–0.97)*	19	0.94 (0.86–0.98)*	19	0.90 (0.76–0.96)*
VE (L/min)	19	0.91 (0.77–0.96)*	19	0.93 (0.84–0.97)*	19	0.92 (0.81–0.97)*
HRpeak (bpm)	19	0.85 (0.66–0.94)*	19	0.88 (0.72–0.95)*	19	0.87 (0.70–0.95)*
RERpeak	19	0.79 (0.53–0.91)*	19	0.60 (0.23–0.82)*	19	0.69 (0.34–0.87)*
Test duration (min)	19	0.92 (0.42–0.98)*	19	0.26 (− 0.04 to 0.67)*	19	0.25 (− 0.03 to 0.66)*
Total work done (kJ)	19	0.97 (0.92–0.99)*	19	0.73 (− 0.07 to 0.93)*	19	0.71 (− 0.08 to 0.92)*
Ventilatory thresholds						
PO at VT1 (W)	16	0.75 (0.43–0.91)*	15	0.56 (− 0.02 to 0.84)*	13	0.68 (0.24–0.89)*
% of POpeak	16	0.30 (− 0.20 to 0.68)	15	0.12 (− 0.36 to 0.57)	13	0.36 (− 0.25 to 0.76)
PO at VT2 (W)	16	0.93 (0.68–0.98)*	17	0.47 (− 0.10 to 0.80)*	15	0.50 (− 0.03 to 0.81)*
% of POpeak	16	0.79 (0.50–0.92)*	17	0.14 (− 0.28 to 0.55)	15	0.02 (− 0.42 to 0.49)
*V*O_2_ at VT1 (L/min)	16	0.61 (0.17–0.85)*	15	0.78 (0.47–0.92)*	13	0.64 (0.16–0.87)*
% of VO_2_peak	16	0.46 (− 0.05 to 0.77)*	15	0.36 (− 0.18 to 0.73)	13	0.13 (− 0.49 to 0.63)
*V*O_2_ at VT2 (L/min)	16	0.84 (0.61–0.94)*	17	0.74 (0.43–0.90)*	15	0.57 (0.14–0.83)*
% of VO_2_peak	16	0.67 (0.27–0.87)*	17	0.57 (0.17–0.82)*	15	0.23 (− 0.16 to 0.62)
HR at VT1 (bpm)	16	0.54 (0.06–0.81)*	15	0.86 (0.65–0.95)*	13	0.66 (0.17–0.88)*
% of HRpeak	16	0.35 (− 0.18 to 0.72)	15	0.78 (0.50–0.93)*	13	0.49 (− 0.09 to 0.82)*
HR at VT2 (bpm)	16	0.61 (0.21–0.84)*	17	0.64 (0.24–0.85)*	15	0.47 (0.00–0.78)*
% of HRpeak	16	0.47 (0.01–0.77)*	17	0.42 (− 0.03 to 0.74)*	15	0.12 (− 0.31 to 0.55)
TWD at VT1 (kJ)	16	0.67 (0.27–0.87)*	15	0.55 (0.02–0.83)*	13	0.61 (0.07–0.87)*
% of TWD at peak	16	0.27 (− 0.26 to 0.67)	15	0.15 (− 0.33 to 0.59)	13	0.30 (− 0.30 to 0.72)
TWD at VT2 (kJ)	16	0.93 (0.80–0.97)*	17	0.49 (0.05–0.78)*	15	0.51 (0.06–0.80)*
% of TWD at peak	16	0.75 (0.43–0.90)*	17	0.22 (− 0.30 to 0.62)	15	0.15 (− 0.34 to 0.59)

*POpeak* peak power output, *VO*_*2*_*peak* peak oxygen uptake, *VCO*_*2*_*peak* peak carbon dioxide production, *VE* minute ventilation, *RERpeak* peak respiratory exchange ratio, *HRpeak* peak heart rate, *TWD* total work done, *VT1* first ventilatory threshold, *VT2* second ventilatory threshold, *ICC* intraclass correlation coefficient, *CI* confidence interval

*ICC is significant at *p* < 0.05

**Fig. 1:**
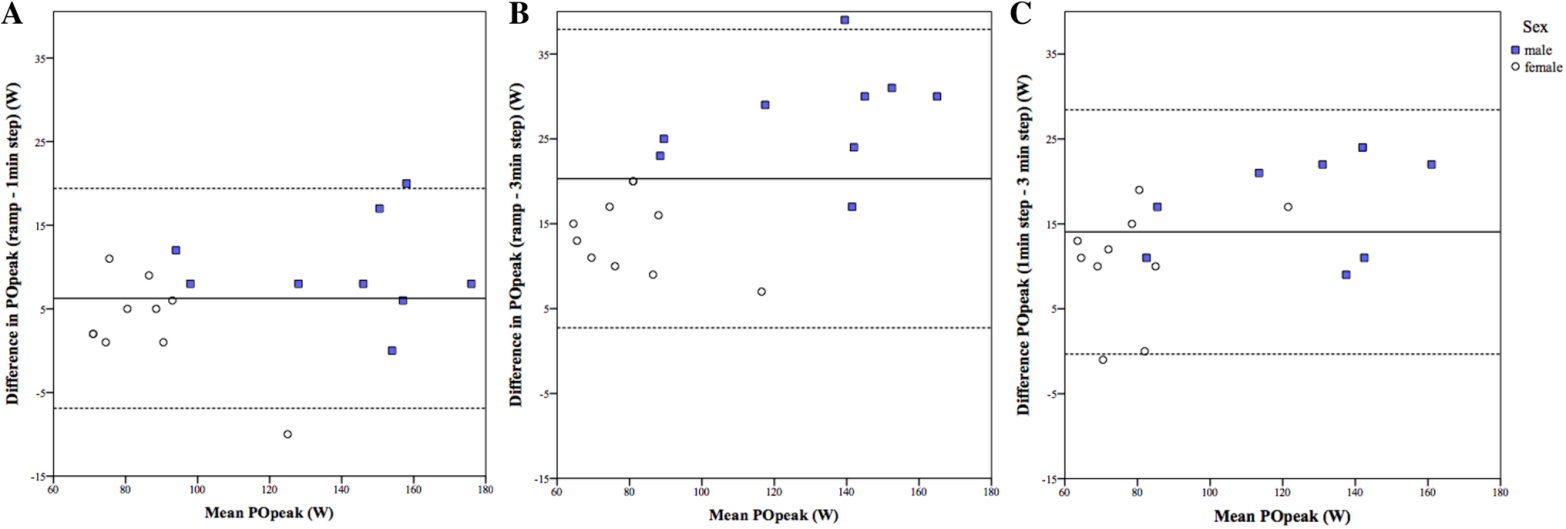
Bland–Altman plot representing absolute agreement. Solid line represents the mean (systematic error), and dotted lines represent mean ± 2SD (95% LoA, random error). Each circle represents a participant (*N* = 19). **a** Absolute agreement of the peak power output (POpeak) between ramp and 1-min stepwise protocol. The intraclass correlation coefficient was very high (0.97), mean difference 6 W, 95% LoA − 7 W to 19 W. **b** Absolute agreement of the POpeak between ramp and 3-min stepwise protocol. The intraclass correlation coefficient was high (0.82), mean difference 20 W, 95% LoA 3–38 W. **c** Absolute agreement of the POpeak between 1-min and 3-min stepwise protocol. The intraclass correlation coefficient was very high (0.90), mean difference 14 W, 95% LoA 0–28 W

At peak level, the relative agreement was high to very high for *V*O_2_, HR, PO, and TWD. For POpeak and TWD, the lower boundaries of the confidence interval were, however, negative for two comparisons. Figure 1 shows the absolute agreement of POpeak among all protocols. The absolute agreement was low with large systematic error and large random error (i.e., wide 95% LoA).

**Fig. 2:**
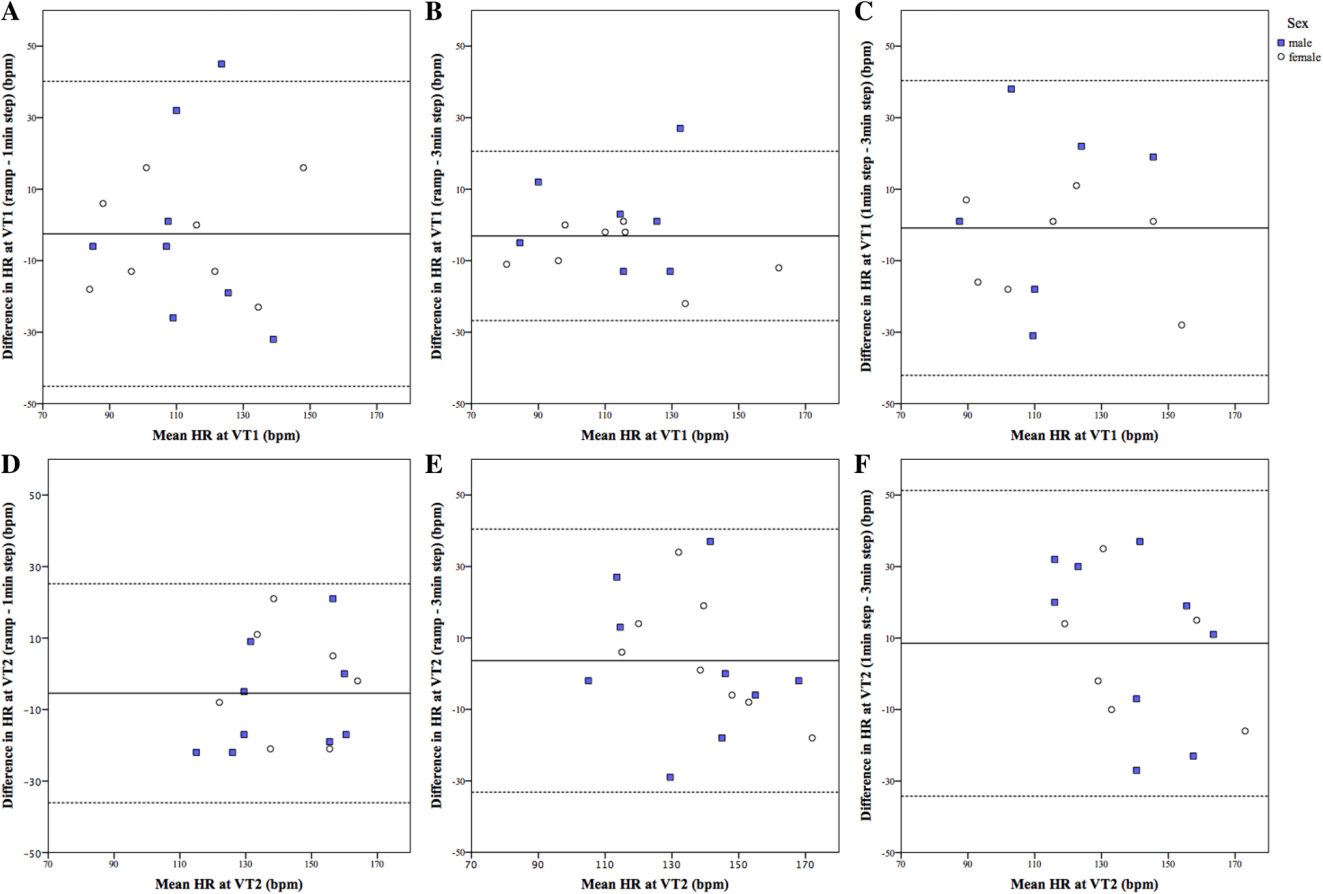
Bland–Altman plot representing absolute agreement. Solid line represents the mean (systematic error), dotted lines represent mean ± 2SD (95% LoA, random error). Each circle represents a participant. **a** Absolute agreement of the heart rate (HR) between ramp and 1-min stepwise protocol (*N* = 16). The intraclass correlation coefficient was moderate (0.54), mean difference 3 bpm, 95% LoA − 45 bpm to 40 bpm. **b** Absolute agreement of the HR between ramp and 3-min stepwise protocol (*N* = 15). The intraclass correlation coefficient was high (0.86), mean difference 3 bpm, 95% LoA − 27 bpm to 21 bpm. **c** Absolute agreement of the HR between 1-min and 3-min stepwise protocol (*N* = 13). The intraclass correlation coefficient was moderate (0.66), mean difference − 1 bpm, 95% LoA − 42 bpm to 40 bpm. **d** Absolute agreement of the HR between ramp and 1-min stepwise protocol (*N* = 16). The intraclass correlation coefficient was moderate (0.61), mean difference − 5 bpm, 95% LoA − 36 bpm to 25 bpm. **e** Absolute agreement of the HR between ramp and 3-min stepwise protocol (*N* = 17). The intraclass correlation coefficient was moderate (0.64), mean difference 4 bpm, 95% LoA − 33 bpm to 40 bpm. **f** Absolute agreement of the HR between 1-min and 3-min stepwise protocol (*N* = 15). The intraclass correlation coefficient was low (0.47), mean difference 9 bpm, 95% LoA − 34 bpm to 51 bpm

At both VTs, the relative agreement was moderate to high for *V*O_2_, low to high for HR, and low to very high for PO and TWD. The agreement of the relative values at both VTs was in general none to low for *V*O_2_, HR, PO, and TWD. Figure 2 shows the absolute agreement of HR at both VTs among all protocols. The absolute agreement was low with small-to-large systematic error and large random error (i.e., wide 95% LoA).

## Discussion

The aim of the present study was to examine the effects of stage duration with a ramp protocol, 1-min stepwise protocol, and 3-min stepwise protocol on PO, *V*O_2,_ and HR at both peak physiological responses and VTs during synchronous arm crank ergometry. The results at peak level demonstrate that PO showed the highest value for the ramp protocol, followed by the 1-min stepwise and 3-min stepwise protocol. At VT1, PO was significantly higher for the ramp protocol compared with the 3-min stepwise protocol, but there was no significant difference between the ramp and 1-min stepwise and the 1-min stepwise and 3-min stepwise protocols. At VT2, PO was significantly higher for both short-stage protocols compared with the 3-min stepwise protocol. No systematic differences for HR and *V*O_2_ were found among protocols, at both VTs and peak level. The relative agreement among protocols varied with low absolute agreement.

### Systematic differences among protocols

The results of the present study are consistent with the previous studies in able-bodied cycling (Amann et al. 2004; Bentley and McNaughton 2003; Bogaard et al. 1996; Zhang et al. 1991; Zuniga et al. 2012) and arm crank ergometry (Smith et al. 2004) that demonstrated no significant differences in *V*O_2_peak and HRpeak among protocols with varying stage duration. The differences in POpeak between the 3-min stepwise protocol and the protocols with short-stage duration (1-min stepwise and ramp) are in line with the previous cycling literature (Amann et al. 2004; Bentley and McNaughton 2003; Bishop et al. 1998; Gullestad et al. 1997; Roffey et al. 2007). Traditionally, short-stage protocols are executed to attain a valid *V*O_2_peak, with the recommendation that test duration should not exceed 12 min (Buchfuhrer et al. 1983). The long-stage protocols, such as the 3-min stepwise protocol, are traditionally executed to attain valid lactate measurements during steady-state conditions to determine a threshold (Bentley et al. 2007). The accompanying recommendation is that increments should be small and step duration at least 3 min (Bishop et al. 1998; Weltman et al. 1990), resulting in a test duration longer than 12 min. The consequence is a different workload over time. Due to the steeper slope in the short-stage protocols, *V*O_2_peak will be reached faster at a higher POpeak within a shorter test duration (Amann et al. 2004; Smith et al. 2006). The lag in *V*O_2_ response that is typically observed in protocols with short-stage duration results in an underestimation of the steady-state *V*O_2_ at that work rate (Davis et al. 1982; Smith et al. 2006). This also explains why studies that set protocols based on time (i.e., all protocols with expected test duration between 8 and 12 min, irrespective of stage duration) do not find a difference in POpeak (Bogaard et al. 1996; Maher and Cowan 2016; Zhang et al. 1991). This, however, does not explain why in the present study, a higher POpeak was found in the ramp protocol compared with the 1-min stepwise protocol, as these protocols were set almost identically (only 2 W difference between protocols after 10-min testing and equal work increments between protocols). POpeak is highly dependent on test design and definition: next to stage duration, work increments and test duration; also starting workload, TWD and definition of POpeak are important aspects (Amann et al. 2004; Bentley et al. 2007; Smith et al. 2006; Zuniga et al. 2012). An explanation for the higher POpeak achieved with the ramp protocol might be the TWD. At a certain similar PO, the TWD was higher for the 1-min stepwise protocol compared with the ramp protocol. In other words, the TWD per minute was higher for the 1-min stepwise protocol compared with the ramp protocol. This higher TWD might lead to fatigue and thus a lower POpeak at the end of the test with shorter test duration (Amann et al. 2004; Smith et al. 2004; Zuniga et al. 2012). The results of the ANOVA in the present study support this: TWD at peak level was not significantly different between the ramp and 1-min stepwise protocol, whereas the corresponding POpeak was significantly lower during the 1-min stepwise protocol. Due to the setup and stepwise character of the 1-min stepwise protocol, participants seem to perform less than with the ramp protocol, whereas in fact, TWD at peak exercise is comparable.

In addition, Smith et al. (2004) argued that motivational factors might also play a role. Participants might use external cues during a stepwise protocol to determine the point at which they finish the test, for example, at the end of a distinct exercise stage, while increments in workload are less perceptible during a ramp protocol. Consequently, smaller increments in workload, for example, 1 W every 5 s instead of distinct 12 W steps every minute, may have less psychological and physiological impact and, therefore, may postpone fatigue and allow participants to reach a higher POpeak (Smith et al. 2004).

Another important aspect is the definition of POpeak. In the present study, POpeak of the ramp protocol was defined as the highest (10 s) PO value at the end of the test, whereas examples exist in which the final 30-s average PO value (Smith et al. 2006) or the mean minute (60 s) ramp power was calculated to be POpeak (Ingham et al. 2013; Smith et al. 2004). If the mean minute ramp power would have been calculated in the present study, POpeak would be 110 ± 36 W and not significantly different from POpeak of the 1-min stepwise protocol. In several studies using ramp protocols, the calculation of POpeak is not clearly stated. This is unfortunate as the example stated above shows that this is a requisite to be able to compare literature.

This is the first study that investigated the effect of stage duration at VTs during synchronous arm ergometry. The results are comparable to the previous literature in able-bodied cycling: no differences in HR and *V*O_2_ at VT1 and VT2 were found among protocols with different stage durations (Bentley and McNaughton 2003; Larson et al. 2015; Zhang et al. 1991), whereas PO at VT1 is significantly lower in tests with longer stage duration (Bentley and McNaughton 2003; Roffey et al. 2007). Although in the present study, there was no systematic difference in the relative PO (i.e., %POpeak) between protocols, we must emphasize that the relative agreement was mostly low or non-existent. Based on the findings in the present study, training zones based on PO at VTs will be at a higher intensity when a short-stage protocol is conducted.

### Agreement among test protocols

In general, the results of the present study demonstrated that the level of relative agreement between the ramp, 1-min stepwise, and 3-min stepwise protocol for PO, HR, and *V*O_2_ was not very promising, since only 12% of ICCs were higher than 0.90. At peak level, the relative agreement between protocols was high to very high for peak values of *V*O_2_, PO, and HR. It must, however, be emphasized that the lower bound of the 95% CI for POpeak was negative in two out of three correlations for POpeak, with low absolute agreement. In addition, there might be a potential effect of heteroscedasticity. The effect is not really evident in Fig. 1, and therefore, studies with more observations should be done to determine this more accurately. Maher and Cowan (2016) compared a 1-min stepwise with a 3-min stepwise protocol during arm crank exercise and reported a relative agreement of 0.96, 0.82, and 0.97 for *V*O_2_peak, HRpeak, and POpeak, respectively. Smith et al. (2004) compared a ramp protocol with a 2-min stepwise protocol during arm crank exercise and reported a relative agreement of 0.67, 0.95, and 0.95 for *V*O_2_peak, HRpeak, and POpeak, respectively. Nevertheless, Smith et al. (2004) concluded that the absolute agreement between protocols was low for all peak outcome measures and, therefore, unacceptable. In the present study, the 95% LoA were also wide, with a low absolute agreement at VT1, VT2, and peak level. In addition, considering biological variation of HR around 5 beats per minute with day-to-day testing (McArdle et al. 2010), it must be concluded that the random variations in the present study are too large to be acceptable. Therefore, it is recommended that these different test protocols in synchronous arm crank exercise should never be used interchangeably within participants to assess cardiorespiratory fitness. In large studies focussing on physical capacity, the use of the same protocol between participants is advised. When this is not possible, multilevel statistical techniques are necessary to correct for possible differences.

### Implications

The results of the present study show that there are systematic differences in PO between protocols at VTs and peak level. Moreover, the absolute agreement in HR at VTs was low due to large random error. Consequently, training zones based on HR or PO will be different among protocols and depending on the chosen protocol. Reviewing the short-stage protocols in the present study, most of the VTs could be determined. However, the non-steady-state character of these protocols results in a certain anaerobic contribution to the PO (Boone and Bourgois 2012). Consequently, training zones for PO based on a ramp protocol or 1-min stepwise protocol will have a higher intensity than zones based on a 3-min stepwise protocol (Bentley et al. 2007). Future studies should investigate which protocol suits best to determine training zones for PO, e.g., whether the ramp protocol gives an overestimation with training zones that are at a too high intensity compared with other protocols in synchronous arm cranking, and whether this might result in overreaching. It is suggested that PO at VTs stemming from ramp and 1-min stepwise protocols could be used as objective means to monitor progress, adaptations, and functional gains associated with training. However, to prevent overestimation, individual training prescription based on PO at VTs would be most secure based on 3-min stepwise protocols, until future studies are performed. An important side note is that protocols with long test duration might not be feasible for certain patient populations with a very low physical capacity or limited arm function. For example, in individuals with a tetraplegia, protocols with short test duration, such as the ramp or 1-min stepwise protocol, might be more appropriate. For these individuals, training intensity based on HR is often not applicable due to the altered sympathetic response to exercise (Valent et al. 2007). It is, therefore, for this population even of more importance to know whether training zones for PO based on short–stage protocols will result in overreaching.

### Limitations

The able-bodied participants in the present study were untrained in arm exercise, unlike wheelchair-bound individuals. We did, however, not find any effects of learning among test one, two, and three. An advantageous aspect of able-bodied participants is that the group is homogeneous and that all participants are physically able to complete all test conditions. The group was relatively small, but comparable to or larger than in the previous studies (Amann et al. 2004; Bentley and McNaughton 2003; Bishop et al. 1998; Bogaard et al. 1996; Gullestad et al. 1997; Roffey et al. 2007; Smith et al. 2004, 2006; Zuniga et al. 2012). Another general limitation of VT determination is that the position of the VT might be different between raters. In the present study, the (relative) interrater reliability was very high, which is acceptable on group level. However, on an individual level in clinical practice, it is advised to evaluate the training zones during training, for example, with a talk test (Kouwijzer et al. 2019). Moreover, in the present study, it was not investigated whether prescribing training intensity based on VT determination is favorable to prescription based on RPE or %POpeak in terms of improvements in cardiorespiratory fitness and in terms of over- or undertraining during upper body exercise. These aspects need to be addressed in future research.

### Future studies

An interesting aspect that was not investigated in the present study is the test–retest reliability of a particular protocol (e.g., the 3-min stepwise protocol) within participants during synchronous arm ergometry. It might be interesting to investigate agreement at VT1, VT2 and peak level with repeated testing of the same exercise protocol in arm exercise, focussing on both trained and untrained individuals and subgroups, such as individuals with paraplegia or tetraplegia. In the light of the present study, it would be interesting to investigate the agreement of PO at both VTs within the 3-min stepwise protocol. It should, however, be considered that protocols with long test duration might be less feasible for individuals with a low physical capacity or limited arm function (e.g., individuals with tetraplegia). Especially, for this population, agreement within short-stage protocols is warranted.

## Conclusion

This study showed that stage duration affects outcomes at both VTs and peak level during synchronous arm crank ergometry in able-bodied participants. No systematic differences for HR and *V*O_2_ were found among protocols. However, PO differed significantly among all protocols at peak level, with the highest value for the ramp protocol, followed by the 1-min stepwise and 3-min stepwise protocol. At VT1, PO was significantly higher for the ramp protocol compared with the 3-min stepwise protocol. At VT2, PO was significantly higher for both short-stage protocols compared with the 3-min stepwise protocol. The relative agreement between protocols varied with low absolute agreement. Consequently, it is recommended that the ramp, 1-min stepwise, and 3-min stepwise arm crank ergometry protocol should never be used interchangeably within persons to assess cardiorespiratory fitness and/or monitor adaptations to training programmes. Furthermore, training prescription based on PO at VTs assessed in short-stage protocols might give an overestimation with training zones that could result in overreaching. Individual training prescription based on PO at VTs would be most secure based on 3-min stepwise protocols; however, protocols with long test duration might not be feasible for certain patient populations with a very low physical capacity. Future studies should pay attention to the effect of stage duration on both peak physiological responses and VTs during arm crank ergometry in subgroups with different abilities and to the consequences of these differences in training zones on training response and overreaching.

## Data Availability

The data sets generated during and/or analysed during the current study are available from the corresponding author on reasonable request.

## Abbreviations

ANOVA: Analysis of variance
CI: Confidence interval
GXT: Graded exercise test
HR: Heart rate
HRpeak: Peak heart rate
ICC: Intraclass correlation coefficient
LoA: Limits of agreement
PAR-Q: Physical Activity Readiness Questionnaire
PO: Power output
POpeak: Peak power output
RER: Respiratory exchange ratio
RERpeak: Peak respiratory exchange ratio
RPE: Ratings of perceived exertion
RPM: Revolutions per minute
SCI: Spinal cord injury
TWD: Total accumulated work done
*V*CO_2_: Carbon dioxide production
VE: Minute ventilation
*V*O_2_: Oxygen uptake
*V*O_2_peak: Peak oxygen uptake
VT: Ventilatory threshold
VT1: First ventilatory threshold
VT2: Second ventilatory threshold
